# The effects of chair yoga practice on stress reduction among high school teachers in Vietnam: a preliminary quasi-experimental study

**DOI:** 10.3389/fpubh.2026.1867357

**Published:** 2026-07-15

**Authors:** Tra Thi Thanh Kieu

**Affiliations:** Department of Psychology, HCMC University of Education, Ho Chi Minh City, Vietnam

**Keywords:** chair yoga, mind-body intervention, school-based intervention, stress reduction, teacher wellbeing

## Abstract

**Background:**

Vietnamese teachers face substantial and persistent work-related stress, yet brief, scalable mind - body interventions have been underexplored in this context. This preliminary study aimed to evaluate the effects of chair yoga practice on stress reduction and the sustainability of benefits with ongoing practice among high school teachers in Vietnam.

**Methods:**

Using a quasi-experimental design, 41 teachers participated in a five-day, school-based chair yoga intervention. Psychological distress was assessed with the DASS-10, and brief written reflections were collected at baseline, immediately after intervention, 2 weeks, and 3 months. Quantitative analyses examined changes in DASS-10 scores and time × practice frequency interactions, while qualitative content analysis explored perceived benefits, challenges, and their evolution across time points.

**Results:**

Distress decreased significantly immediately after the intervention [Cohen’s d = 0.779, 95% CI (0.425, 1.125)]. Over time, a clear dose–response pattern emerged: teachers who practiced chair yoga regularly (approximately 1–2 times per week) showed the largest and most sustained reductions in distress, whereas those who rarely or never practiced demonstrated partial loss or rebound of symptoms, reflected in large time × practice interaction effects at 2 weeks (ηp^2^ = 0.382) and 3 months (ηp^2^ = 0.537). Qualitative findings paralleled these trajectories: initial reflections emphasized relaxation and stress relief alongside physical and scheduling challenges; by 2 weeks, participants increasingly described transformation experiences and greater bodily comfort; by 3 months, they reported a more stable profile of psychological and physical benefits, despite persistent external barriers to practice.

**Conclusion:**

A brief chair yoga intervention appears feasible and acceptable in a Vietnamese high school and can rapidly reduce teacher distress, with regular ongoing practice associated with more durable benefits and the development of chair yoga as a habitual coping strategy. However, the quasi-experimental, single-site design, modest sample, attrition, end-of-year timing of the final follow-up, and coarse self-reported adherence measures mean that these findings should be considered preliminary. Even so, the results contribute to growing evidence that low-cost, school-based mind–body interventions may support teacher wellbeing and highlight the need for controlled trials and implementation research in diverse educational settings.

## 1 Introduction

Teaching is widely recognized as a profession that can provide satisfaction and a sense of fulfillment; however, it is also associated with considerable psychological and physical demands, often leading to emotional exhaustion (1–3). Since the late 20th century, work-related stress, fatigue, and burnout among teachers have become prominent topics of global research (4, 5). A growing body of literature consistently identifies teaching as a high-risk profession for burnout across diverse contexts (4–9). Across educational systems, teachers report high levels of psychological strain associated with increasing administrative demands, curriculum reforms, performance accountability, classroom management challenges, and role ambiguity (1, 10–12). Chronic exposure to occupational stress not only compromises teachers’ mental health but also negatively affects instructional quality, teacher retention, and student outcomes (13, 14).

In Vietnam, the teaching profession has undergone rapid transformation in recent years due to curriculum reforms, digital integration, competency-based education regulations, and increased accountability standards (15, 16). These changes have been associated with increased workload and time pressure, contributing to high levels of psychological distress among teachers, including anxiety and emotional exhaustion (17–24). At the same time, evidence indicates that teachers also have substantial needs for support across professional development, psychological, and mental health care (25). Despite these challenges, psychological and mental health support services within Vietnamese remain quite limited, particularly for educators, and school-based wellbeing programs are not yet systematically implemented (26, 27). This gap highlights the necessity for low-cost interventions that teachers can easily incorporate into their daily routines.

Mind–body interventions, including yoga, have become more popular as accessible and practical approaches for managing stress across diverse groups. Yoga encompasses physical movement, breathing exercises, and concentrated mindfulness (28, 29). The combination of these aspects can affect stress responses through both physiological and psychological mechanisms, resulting in a reduction in stress, anxiety, and emotional suffering among diverse populations (29–34). A substantial body of research has examined yoga- and mindfulness-based interventions for teachers, commonly involving structured practices such as breathing exercises, body scans, and mindful movement or yoga techniques (35). Findings from targeted yoga and mindfulness programs frequently indicate improvement in present-moment awareness and nonjudgmental observation of internal experiences, correlating with decreases in stress, anxiety, emotional exhaustion, and burnout (36–46). Additionally, these programs are associated with improved teaching efficacy (39, 45), personal accomplishment, job and life satisfaction (37, 39, 41, 47), affect (37, 45), and overall wellbeing (38–40, 42–44, 46–48).

However, the applicability of these findings to the Vietnamese context remains uncertain. Most existing studies are conducted in Western countries or India, where school systems, cultural attitudes towards yoga, and working conditions differ markedly from those in Vietnam. This situation limits the direct transferability of findings and underlines the need for context-specific research with Vietnamese teachers. Moreover, many yoga-based interventions require mats, open floor space, and time outside regular teaching hours (45, 49), which may not be feasible in Vietnam, where teachers often have limited free time and work in space-constrained environments. Chair yoga, defined as yoga practiced while seated on or using a chair for support (50), offers a potentially suitable alternative. It has been successfully used in other populations with space, mobility, or time constraints and can improve mood, fatigue, and stress-related outcomes (50–56). Nevertheless, no studies to date have examined chair yoga as a prevention measure for stress among Vietnamese teachers.

In response to these gaps, the present study, titled “The Effects of Chair Yoga Practice on Stress Reduction Among High School Teachers in Vietnam: A Preliminary Quasi-Experimental Study” is proposed. Drawing on evidence that yoga and mindfulness practice can reduce educator stress (37–40, 42–47, 57, 58), and that chair yoga is a feasible, low cost format in constrained settings (50–53), the study will implement a structured chair yoga program that can be practiced in classrooms, staff rooms or at home without any special equipment. The intervention will focus on simple seated postures, gentle stretching, and breathing exercises that fit into teachers’ daily schedules. By implementing a structured chair yoga program tailored to the school context, this study aims to evaluate whether chair yoga practice can significantly reduce stress among Vietnamese high school teachers. Furthermore, the study illustrates how a brief, low-intensity, yoga-based mind–body intervention can be adapted to real-world occupational conditions and integrated into daily routines, positioning chair yoga not only as a stress-management tool for educators but also as a practical model of workplace health promotion in a resource-constrained context.

## 2 Methodology

### 2.1 Study design

The study was conducted in a private semi-boarding high school in Da Lat City, Lam Dong province, Vietnam, from November 2023 to April 2024 and comprised three main phases:

#### 2.1.1 Phase 1

Recruitment and baseline assessment (Final week of November 2023): During the last week of November 2023, with approval from the school administration, a call for participation was issued to invite high school teachers to join the study. We enrolled teachers that met the inclusion and exclusion criteria of the study as stated in Table 1. As part of the recruitment process, study invitations were distributed and participant information was collected to verify eligibility based on the inclusion and exclusion criteria. A total of 63 eligible participants met the criteria, provided informed consent, and completed the baseline assessment (t_0_), which included the Depression, Anxiety, and Stress Scale-10 (DASS-10) (59) to assess overall psychological distress.

**Table 1:** The inclusion and exclusion criteria of the study.

Criterion	Description
Inclusion	Full-time teachers and staff members currently working at the school; Not having practiced yoga regularly for at least the past 3 months; No current diagnosis of neurological or psychiatric disorders, or any acute medical condition that contraindicates yoga practice.
Exclusion	Pregnancy; Diagnosis of any neurological or psychiatric disorders, or any acute medical condition that contraindicates yoga practice; Have experienced trauma or bereavement within the past month Currently practicing yoga regularly Currently engaging in other mind–body interventions.

#### 2.1.2 Phase 2

Chair yoga training: From 6 to 10 December 2023, participants attended a five-day chair yoga program, consisting of one 45-min session per day held in the school’s staff meeting room after working hours at 4:30 p.m. The chair yoga sessions were designed and led by a certified, experienced yoga instructor focusing on gentle stretching, breathing exercises, and relaxation techniques. The program was tailored to the school context, emphasizing stress reduction and sustainable self-care practices suitable for teachers’ working conditions. Immediately after completing the five-day chair yoga training (on 10 December 2023), participants completed a post-intervention survey (t_1_).

#### 2.1.3 Phase 3

**Figure 1:**
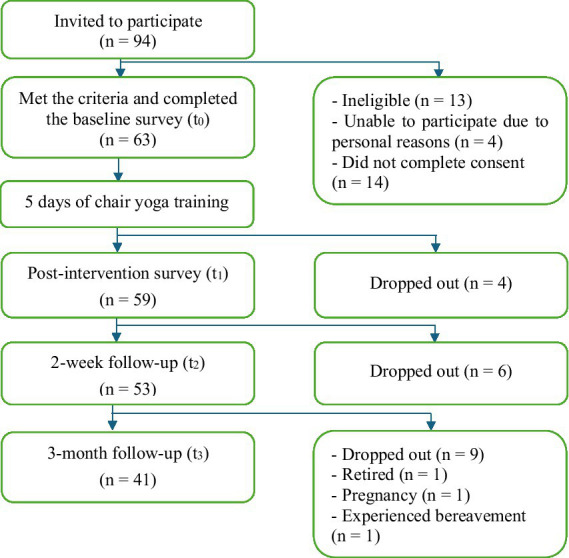
The intervention study participant flow diagram.

Independent practice and follow-up (December 2023–April 2024): Following the training, teachers were encouraged to continue practicing chair yoga independently as part of their daily routines. During the follow-up phase, participants’ health status and related conditions were also monitored. Specifically, participants were advised to seek appropriate support if they developed health issues or experienced potentially traumatic events. Follow-up surveys were conducted at 2 weeks (t_2_) and 3 months (t_3_) post-training, again using the DASS-10 to evaluate sustained changes in distress levels. Qualitative reflections collected during these follow-ups provided further insights into participants’ continued engagement, perceived stress reduction, and the integration of chair yoga into everyday life. At the 2-week follow-up, 6 participants had dropped out, and 53 completed the survey. By the three-month follow-up, an additional 9 participants had dropped out, 1 had retired, and 2 participants, one who became pregnant and another who experienced bereavement, were excluded, resulting in a final sample of 41 participants who completed the study. The mean age of participants was 35.15 years (SD = 8.69), with similar age distributions observed between females (*M* = 35.54, SD = 8.31) and males (*M* = 34.47, SD = 9.58). Briefly, the intervention study participant flow diagram is presented in Figure 1.

### 2.2 Intervention: chair yoga program

To accommodate the school’s facilities and ensure convenience for participants, the yoga program was designed as chair yoga, rather than mat-based yoga. The intervention program was structured as a three-stage progression designed to gradually regulate stress responses. A detailed description of the 5-day chair yoga program is provided in Supplementary material 1. After the training, participants were encouraged to continue practicing at least once per week, supported by a pictorial guidebook for independent use.

#### 2.2.1 Stage 1—preparatory phase

The first stage consisted of gentle joint-mobilization exercises drawn from the Pawanmuktasana series, a group of preparatory movements in Hatha yoga commonly used to increase circulation, release muscular tension, and improve joint mobility and body awareness (60–62). Practiced with slow, synchronized breathing, these low-amplitude repetitions were intended to enhance proprioceptive feedback to the central nervous system, prepare balance and coordination systems for subsequent postures, and promote relaxation and focused attention to help down-regulate stress-related physiological arousal.

#### 2.2.2 Stage 2—adjustment phase

The second stage used standing and single-leg balance postures (e.g., tree pose, warrior III,…) that require continuous postural adjustment, thereby engaging vestibular and proprioceptive feedback and core stabilizing muscles, while sustained attentional focus during balancing may reduce rumination and foster present-moment, body-centered awareness (29, 63).

#### 2.2.3 Stage 3—cooling down phase

The final stage comprised slow, regulated pranayama techniques (e.g., full yogic breathing, sectional breathing, Ujjayi, and Kapalabhati) intended to modulate autonomic activity; slow, controlled breathing patterns have been shown to enhance parasympathetic tone, improve autonomic balance, and reduce stress-related cardiovascular reactivity, while breath-focused attention supports calmer, more stable mental states (29, 64–66).

### 2.3 Measures

#### 2.3.1 DASS-10

Halford and Frost (59) developed the DASS-10 as a concise derivative of the original 42-item Depression, Anxiety, and Stress Scales (DASS-42) proposed by Lovibond and Lovibond (67). The 10-item version is designed to assess overall psychological distress. In the present study, the DASS-10 (Vietnamese version translated by Nguyen Thi Thanh Huong, Dinh Xuan Dai, Nguyen Thanh Thao, Pham Cam Anh, and Nguyen Minh Van) was used as the main tool to evaluate changes in participants’ overall distress following the chair yoga practice. Internal consistency in the current sample was high, with reliability coefficients ranging from 0.86 to 0.92, indicating excellent scale reliability.

#### 2.3.2 Practice adherence and frequency

In the follow-up surveys, participants were asked whether they continued practicing the exercises (at the 2-week and 3-month follow-ups) and about the frequency of their practice (at the 3-month follow-up).

#### 2.3.3 Qualitative evaluation of participant experience

For the qualitative component, open-ended responses about participants’ feedback on chair yoga practice were collected at post-intervention (t_1_), 2-week follow-up (t_2_), and three-month follow-up (t_3_), and were analyzed using a frequency-based content analysis approach. At each time point, participants responded to the following question: “*Please describe any feelings you have noticed in your body and mind since practicing these exercises during [the past 5 days (t_1_)/the last 2 weeks (t_2_)/the last 3 months (t_3_)]*.” Participants were also invited to share additional comments or reflections related to their practice. Responses were written in Vietnamese and subsequently coded for analysis. Key phrases were extracted, categorized into themes, and quantified using frequencies (n) and proportions (%) for each theme across time points.

## 3 Results

### 3.1 The quantitative results

#### 3.1.1 Baseline characteristics of participants

The participants’ characteristics and baseline DASS-10 score are presented in Table 2. The mean age of participants was 35.15 years (SD = 8.69). On average, participants had 12.02 years of teaching experience (SD = 8.64). Participants taught a range of subjects, including natural sciences (26.8%), social sciences (29.3%), foreign languages (24.4%), and other subjects (19.5%), reflecting a diverse mix of disciplines. Teachers at this institution were assigned a teaching load of 17 periods per week, in accordance with the standard workload regulations stipulated in Circular No. 28/2009/TT-BGDDT (68). At baseline, the mean DASS-10 score for the overall sample was 6.93, 95% CI [5.41, 8.45], SD = 4.81 indicating a level of psychological distress approaching the moderate range.

**Table 2:** Participant characteristics and baseline DASS-10 scores.

Variable	Value
Gender	*n* (%)
Male	15 (36.59%)
Female	26 (63.41%)
Age (years)	M_age_ = 35.15; SD_age_ = 8.69
Teaching experience (years)	M_teaching experience_ = 12.02; SD _teaching experience_ = 8.64
Teaching subjects	*n* (%)
Natural sciences	11 (26.8%)
Social sciences	12 (29.3%)
Foreign languages	10 (24.4%)
Other subjects	8 (19.5%)
Baseline DASS-10 score (t_0_)	M_DASS-10(baseline)_ = 6.93; 95% CI _DASS-10(baseline)_ [5.41, 8.45]; SD_DASS-10(baseline)_ = 4.81

#### 3.1.2 Post-intervention results

The pre-post intervention differences in DASS-10 scores among participants are reported in Table 3. A paired-samples t-test revealed a significant reduction in DASS-10 scores following the intervention. The DASS-10 score decreased from 6.93, 95% CI [5.41, 8.45], SD = 4.81 at baseline to 4.39, 95% CI [3.15, 5.63], SD = 3.92 post-intervention, representing a statistically significant improvement in psychological distress, *t* (40) = 4.99, *p* < 0.001. The effect size, Cohen’s *d* = 0.779, 95% CI [0.425, 1.125], indicated a meaningful stress reduction after the chair yoga program intervention.

**Table 3:** Pre–post intervention differences in DASS-10 scores.

Variable	Pre-intervention (t_0_)			Post-intervention (t_1_)			Paired sample t-test			
	Mean	95% CI for Mean (Lower–Upper)	SD	Mean	95% CI for Mean (Lower–Upper)	SD	*t*	*p*-value	Cohen’s *d*	95% CI for Cohen’s *d* (Lower–Upper)
DASS-10	6.93	5.41–8.45	4.81	4.39	3.15–5.63	3.92	4.988	<0.001	0.779	0.425–1.125

#### 3.1.3 The 2-week follow-up results

**Figure 2:**
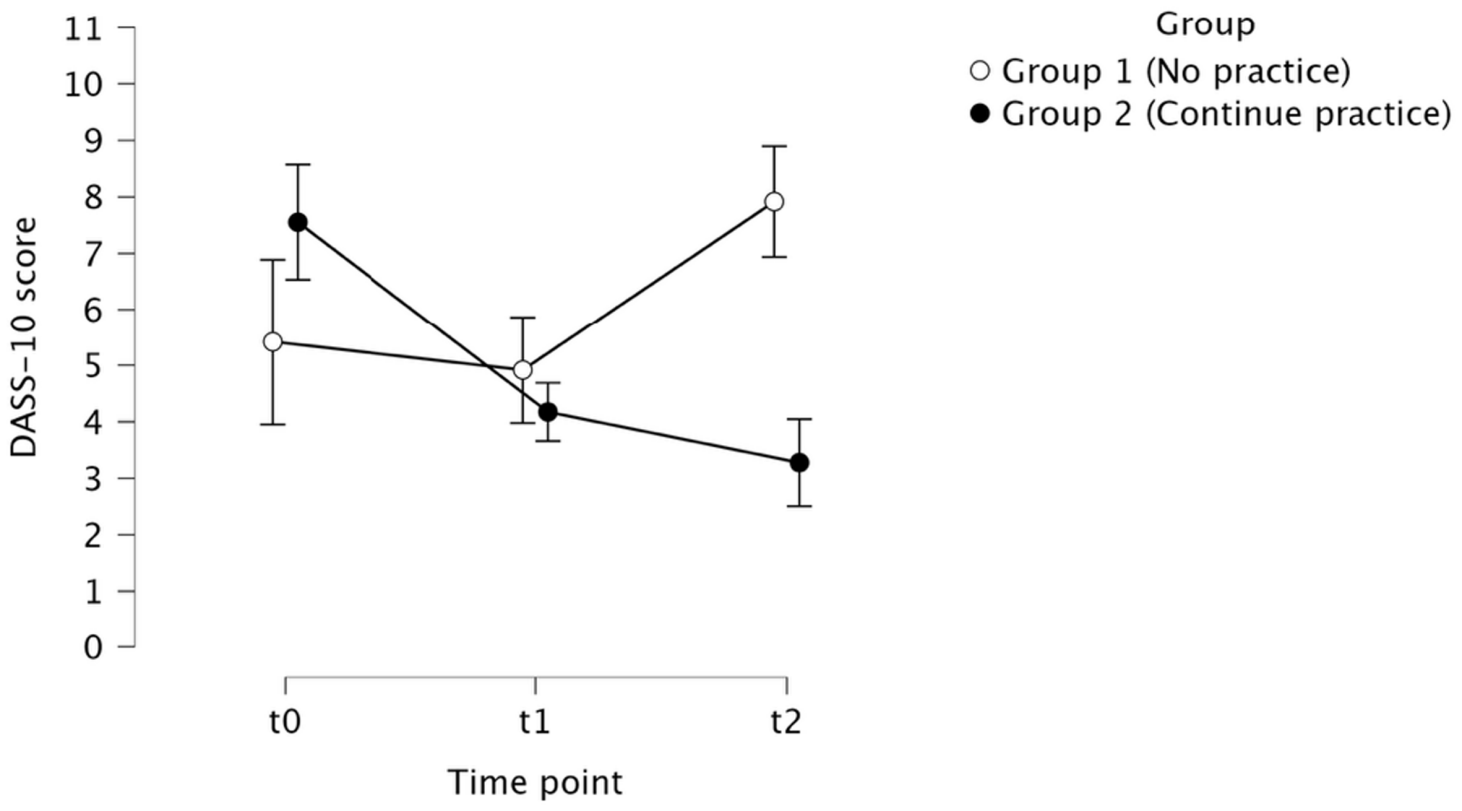
Discriptive plots of DASS-10 score changes across time at 2-week follow up survey by practice groups. Note: Mean DASS-10 scores at baseline (t_0_), post-intervention (t_1_), and 2-week follow-up (t_2_) are plotted separately for teachers who reported no practice (Group 1, *n* = 12), and and those who continued practicing (Group 2, *n* = 29) during the follow-up period. Error bars indicate 95% confidence intervals.

At the 2-week follow-up, participants were categorized into two groups based on practice behavior: those who did not practice (*n* = 12) and those who continued practicing at least once per week (*n* = 29). The results by practice group are presented in Table 4 and Figure 2. Participants who continued practicing showed a sustained and progressive reduction in distress, with mean scores decreasing from baseline (t_0_: *M* = 7.55, SD = 4.77) to post-intervention (t_1_: *M* = 4.17, SD = 3.88), and further to 2 weeks (t_2_: *M* = 3.28, SD = 2.91). In contrast, those who did not continue their practice demonstrated a different pattern. Although a slight improvement was observed immediately after the intervention (t_0_: *M* = 5.42, SD = 4.76; t_1_: *M* = 4.92, SD = 4.12), distress increased markedly at the 2-week follow-up (t_2_: *M* = 7.92, SD = 4.42), exceeding baseline scores.

**Table 4:** Changes in DASS-10 scores at pre-intervention, post-intervention, and 2-week follow-up by practice groups.

Group	Pre-intervention (Mean ± SD)	Post-intervention (Mean ± SD)	After 2-week (Mean ± SD)
No practice during 2 weeks (*n* = 12)	5.42 ± 4.76	4.92 ± 4.12	7.92 ± 4.42
Ongoing practice during 2 weeks (*n* = 29)	7.55 ± 4.77	4.17 ± 3.88	3.28 ± 2.91
Total (*n* = 41)	6.93 ± 4.81	4.39 ± 3.92	4.63 ± 3.99

Repeated measure ANOVA results for DASS-10 scores at 2-week follow-up by practice groups are reported in Table 5. A repeated-measures ANOVA indicated a significant main effect of time, F(1.365, adjusted df) = 7.857, *p* = 0.003, ηp^2^ = 0.168, and a highly significant time × practice group interaction, F(1.365, adjusted df) = 24.093, *p* < 0.001, ηp^2^ = 0.382. The large interaction effect suggests that continued practice played a critical role in shaping short-term outcomes. The between-subjects effect of practice group was not significant, *F* (1, 39) = 0.715, *p* = 0.403, ηp^2^ = 0.018, indicating that differences were primarily driven by changes over time rather than baseline group differences.

**Table 5:** Repeated measures ANOVA results for DASS-10 scores at 2-week follow-up by practice groups.

Cases	*F*	df	*p*-value	η_p_^2^
Within subjects^*^				
Time	7.857	1.365	0.003	0.168
Time × practice groups^**^	24.093	1.365	<0.001	0.382
Between subjects				
Practice groups	0.715	1	0.403	0.018

*Mauchly test confirmed the assumption of sphericity was violated and ε < 0.75 hence Greenhouse–Geisser correction was used.

**Practice groups were categorized as follows: Group 1 (*n* = 12): Participants who did not practice chair yoga during the 2-week follow-up period; Group 2 (*n* = 29): Participants who continued practicing chair yoga at least once per week during the 2-week follow-up period.

*Post hoc* comparisons (Table 6) examining differences between the no-practice group and the continued-practice group across time points revealed no significant group differences at t_0_ or t_1_. At baseline, the no-practice group showed slightly lower scores than the continued-practice group, although this difference was not statistically significant [mean difference = −2.135, 95% CI for mean difference (−5.444, 1.174), *t* (39) = − 1.305, *p*_Bonf_ = 0.200, Cohen’s *d* = −0.523, 95% CI for Cohen’s *d* (−1.791, 0.744)]. Similarly, at t_1_, no significant difference was observed between the groups [mean difference = 0.744, 95% CI for mean difference (−1.999, 3.488), *t* (39) = 0.549, *p*_Bonf_ = 0.586, Cohen’s *d* = 0.182, 95% CI for Cohen’s *d* (−0.859, 1.224)]. However, at t_2_, the no-practice group reported significantly higher scores than the continued-practice group [mean difference = 4.641, 95% CI for mean difference (2.275, 7.006), *t* (39) = 3.968, *p*_Bonf_ < 0.001, Cohen’s *d* = 1.137, 95% CI for Cohen’s *d* (0.155, 2.120)]. These findings suggest that group differences became apparent over time, with the no-practice group showing significantly higher scores than the continued-practice group after a 2-week follow-up.

**Table 6:** *Post hoc* pairwise comparisons of stress scores by practice group across 3 time points.

Time point			Mean difference	95% CI for mean difference		SE	df	*t*	Cohen’s *d*	95% CI for Cohen’s *d*		*p*_Bonf_
				Lower	Upper					Lower	Upper	
t_0_	Group 1	Group 2	−2.135	−5.444	1.174	1.636	39	−1.305	−0.523	−1.791	0.744	0.200
t_1_		Group 2	0.744	−1.999	3.488	1.356	39	0.549	0.182	−0.859	1.224	0.586
t_2_		Group 2	4.641	2.275	7.006	1.169	39	3.968	1.137	0.155	2.120	< 0.001^***^

***p < 0.001.

Practice groups were categorized as follows: Group 1 (*n* = 12): participants who did not practice chair yoga during the 2-week follow-up period; Group 2 (*n* = 29): participants who continued practicing chair yoga at least once per week during the 2-week follow-up period.

#### 3.1.4 The 3-month follow-up results

**Figure 3:**
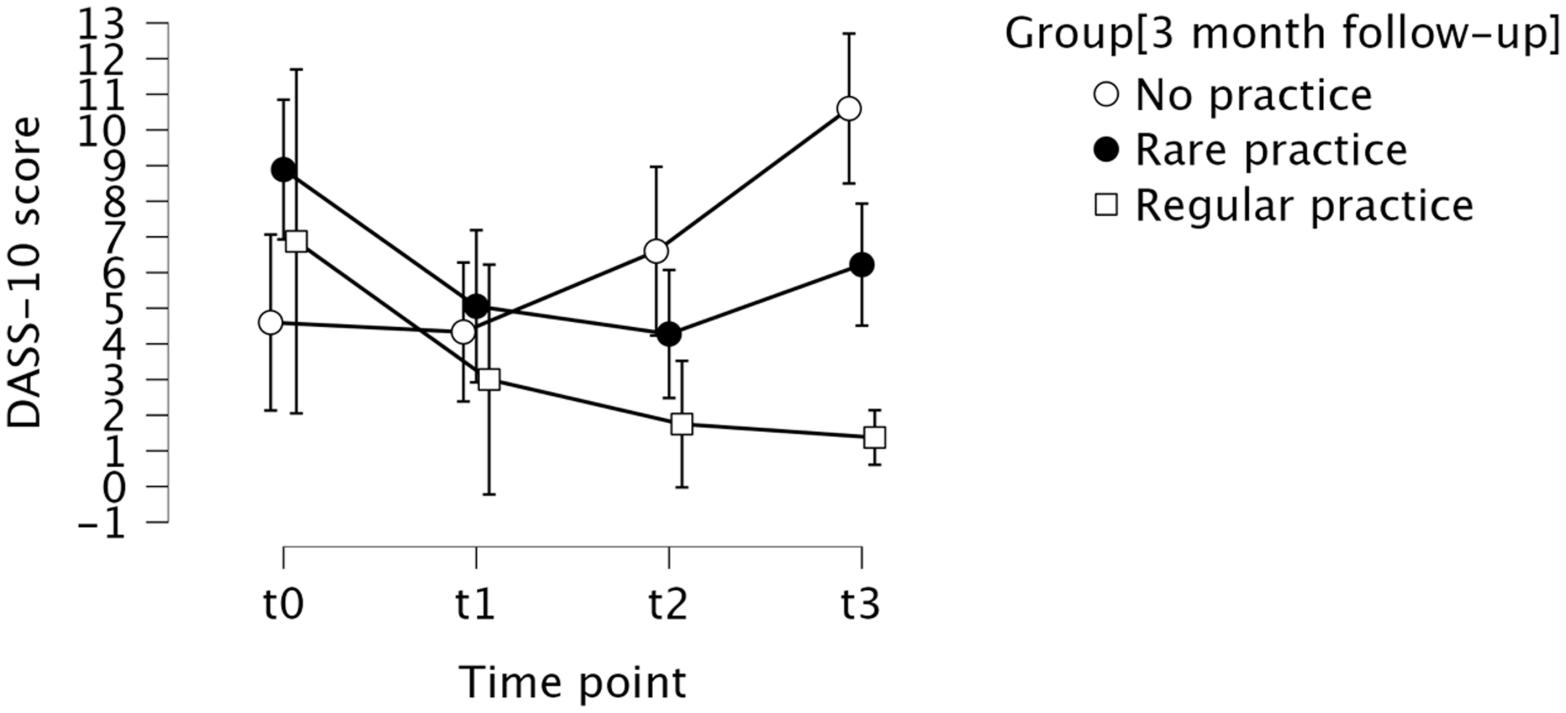
Discriptive plots of DASS-10 score changes across time at 3-month follow up survey by practice groups. Note: Mean DASS-10 scores at baseline (t_0_), post-intervention (t_1_), 2-week follow-up (t_2_), and three-month follow-up (t_3_) are plotted separately for teachers who reported no practice (*n* = 15), rare practice (1–2 times per month) (*n* = 18), and regular practice (1–2 times per week) (*n* = 8) during the follow-up period. Error bars indicate 95% confidence intervals.

At the three-month follow-up, participants were categorized into three groups: no practice (*n* = 15), rare practice (1–2 times/month; *n* = 18), and regular practice (1–2 times/week; *n* = 8). Distinct longitudinal patterns emerged (Table 7 and Figure 3):

- Participants with no practice showed a progressive increase in distress over time, with scores rising from post-intervention (t_1_: *M* = 4.33, SD = 3.52) to 2 weeks (t_2_: *M* = 6.60, SD = 4.42) and sharply to 3 months (t_3_: *M* = 10.60, SD = 3.79), substantially exceeding baseline levels (t_0_: *M* = 4.60, SD = 4.45).
- Those with rare practice maintained initial improvements (t_0_: *M* = 8.89, SD = 3.94; t_1_: *M* = 5.06, SD = 4.29; t_2_: *M* = 4.28, SD = 3.61), but experienced a partial rebound at 3 months (t_3_: *M* = 6.22, SD = 3.44), indicating attenuation of intervention effects.
- Participants who regularly practiced demonstrated sustained and cumulative benefits, with distress steadily decreasing from baseline (t_0_: *M* = 6.88, SD = 5.77) to post-intervention (t_1_: *M* = 3.00, SD = 3.85), further at 2 weeks (t_2_: *M* = 1.75, SD = 2.12), and reaching minimal levels at 3 months (t_3_: *M* = 1.38, SD = 0.92).

**Table 7:** Changes in DASS-10 scores at pre-intervention, post-intervention, 2-week and 3-month follow-up by practice groups.

Group	Pre-intervention (Mean ± SD)	Post-intervention (Mean ± SD)	After 2 weeks (Mean ± SD)	After 3 months (Mean ± SD)
No practice during 3 months (*n* = 15)	4.60 ± 4.45	4.33 ± 3.52	6.60 ± 4.42	10.60 ± 3.79
Rare practice (1–2 times per month) during 3 months (*n* = 18)	8.89 ± 3.94	5.06 ± 4.29	4.28 ± 3.61	6.22 ± 3.44
Regular practice (1–2 times per week) during 3 months (*n* = 8)	6.88 ± 5.77	3.00 ± 3.85	1.75 ± 2.12	1.38 ± 0.92
Total (*n* = 41)	6.93 ± 4.81	4.39 ± 3.92	4.63 ± 3.99	6.88 ± 4.65

Repeated-measures ANOVA (Table 8) revealed a significant main effect of time, *F* (1.787, adjusted df) = 16.75, *p* < 0.001, ηp^2^ = 0.306, and a very large time × practice group interaction, *F* (1.787, adjusted df) = 22.00, *p* < 0.001, ηp^2^ = 0.537. The between-subjects effect of the practice groups approached significance, *F* (2, 38) = 2.541, *p* = 0.092, ηp^2^ = 0.118, suggesting a moderate but non-significant overall difference in distress levels across groups.

**Table 8:** Repeated measures ANOVA results for DASS-10 scores at 3-month follow-up by practice groups.

Cases	*F*	df	*p*-value	η_p_^2^
Within subjects^*^				
Time	16.75	1.787	< 0.001	0.306
Time × practice groups^**^	22.00	1.787	<0.001	0.537
Between subjects				
Practice groups	2.541	2	0.092	0.118

^*^Mauchly test confirmed the assumption of sphericity was violated and ε < 0.75 hence Greenhouse–Geisser correction was used.

^**^Practice groups were categorized as follows: Group 1 (*n* = 15): participants who did not practice chair yoga during the three-month follow-up period; Group 2 (*n* = 18): participants who rarely practiced chair yoga (1–2 times per month) during the three-month follow-up period; Group 3 (*n* = 8): participants who regularly practiced chair yoga (1–2 times per week) during the three-month follow-up period.

*Post hoc* tests (Table 9) showed that, at baseline (t_0_), the no-practice group reported lower distress than the rare-practice group, mean difference = −4.289, 95% CI for mean difference [−8.241, −0.336], t (38) = − 2.718, *p*_Bonf_ = 0.030, Cohen’s *d* = −1.107, 95% CI for Cohen’s *d* [−2.669, 0.456]. No other baseline contrasts involving the regular-practice group were significant (all 95% CIs for mean difference included zero, all *p*_Bonf_ > 0.05). At t_1_, none of the pairwise comparisons were significant (all *p*_Bonf_ > 0.05). At t_2_, participants in the no-practice group reported significantly higher scores than those in the regular-practice group [mean difference = 4.850, 95% CI for mean difference (0.838, 8.862), *t* (38) = 3.028, *p*_Bonf_ = 0.013, Cohen’s *d* = 1.252, 95% CI for Cohen’s *d* (−0.351, 2.855)]. However, the difference between the no-practice and rare-practice groups was not significant [mean difference = 2.322, 95% CI (−0.882, 5.526), *p*_Bonf_ = 0.232], nor was the difference between the rare-practice and regular-practice groups [mean difference = 2.528, 95% CI (−1.366, 6.422), *p*_Bonf_ = 0.337].

**Table 9:** *Post hoc* pairwise comparisons of stress scores by practice group across 4 time points.

Time point			Mean difference	95% CI for mean difference		SE	df	t	Cohen’s *d*	95% CI for Cohen’s *d*		*p*_Bonf_
				Lower	Upper					Lower	Upper	
t_0_	Group 1	Group 2	−4.289	−8.241	−0.336	1.578	38	−2.718	−1.107	−2.669	0.456	0.030^*^
		Group 3	−2.275	−7.225	2.675	1.976	38	−1.151	−0.587	−2.471	1.297	0.771
	Group 2	Group 3	2.014	−2.790	6.818	1.918	38	1.050	0.520	−1.306	2.346	0.901
t_1_	Group 1	Group 2	−0.722	−4.173	2.729	1.378	38	−0.524	−0.186	−1.491	1.118	1.000
		Group 3	1.333	−2.988	5.655	1.725	38	0.773	0.344	−1.293	1.982	1.000
	Group 2	Group 3	2.056	−2.139	6.250	1.675	38	1.227	0.531	−1.068	2.129	0.682
t_2_	Group 1	Group 2	2.322	−0.882	5.526	1.279	38	1.815	0.599	−0.636	1.834	0.232
		Group 3	4.850	0.838	8.862	1.602	38	3.028	1.252	−0.351	2.855	0.013^*^
	Group 2	Group 3	2.528	−1.366	6.422	1.555	38	1.626	0.652	−0.843	2.147	0.337
t_3_	Group 1	Group 2	4.378	1.507	7.249	1.146	38	3.819	1.130	−0.053	2.313	0.001^**^
		Group 3	9.225	5.629	12.82	1.436	38	6.426	2.381	0.695	4.067	< 0.001^***^
	Group 2	Group 3	4.847	1.357	8.337	1.393	38	3.479	1.251	−0.167	2.669	0.004^**^

^*^p < 0.05, ^**^p < 0.01, ^***^p < 0.001.

Practice groups were categorized as follows: Group 1 (*n* = 15): participants who did not practice chair yoga during the three-month follow-up period; Group 2 (*n* = 18): participants who rarely practiced chair yoga (1–2 times per month) during the three-month follow-up period; Group 3 (*n* = 8): participants who regularly practiced chair yoga (1–2 times per week) during the three-month follow-up period.

By 3 months (t_3_), the no-practice group showed substantially higher distress than the regular-practice group, with a mean difference of 9.225, 95% CI for mean difference [5.629, 12.82], t (38) = 6.43, *p*_Bonf_ < 0.001, Cohen’s *d* = 2.381, 95% CI for Cohen’s *d* [0.695, 4.067], indicating a large effect. The rare-practice group also reported significantly higher distress than the regular-practice group at t_3_, mean difference = 4.847, 95% CI for mean difference [1.357, 8.337], t (38) = 3.48, *p*_Bonf_ = 0.004, Cohen’s *d* = 1.251, 95% CI for Cohen’s *d* [−0.167, 2.669], suggesting a substantial but less precise effect. In addition, the no-practice group demonstrated significantly higher distress than the rare-practice group, mean difference = 4.378, 95% CI for mean difference [1.507, 7.249], t (38) = 3.82, *p*_Bonf_ = 0.001, Cohen’s *d* = 1.130, 95% CI for Cohen’s *d* [−0.053, 2.313]. Together, these findings suggest a graded pattern in which greater engagement in practice was associated with progressively lower levels of stress over time.

### 3.2 The qualitative results

**Figure 4:**
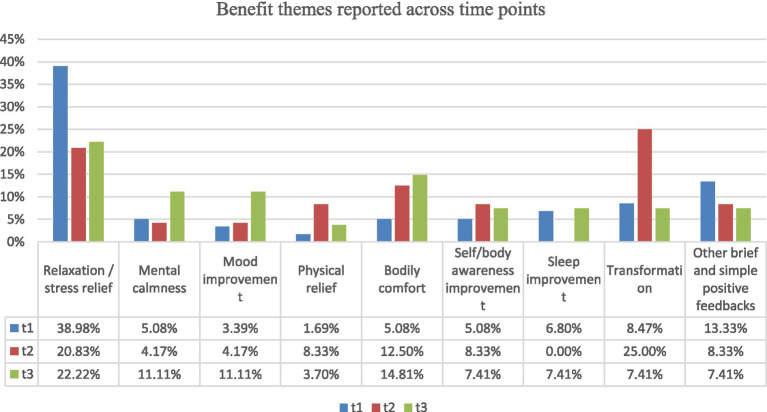
Distribution of perceived chair yoga benefits at post-intervention (t_1_), 2-week (t_2_), and three-month (t_3_) follow-up.

**Figure 5:**
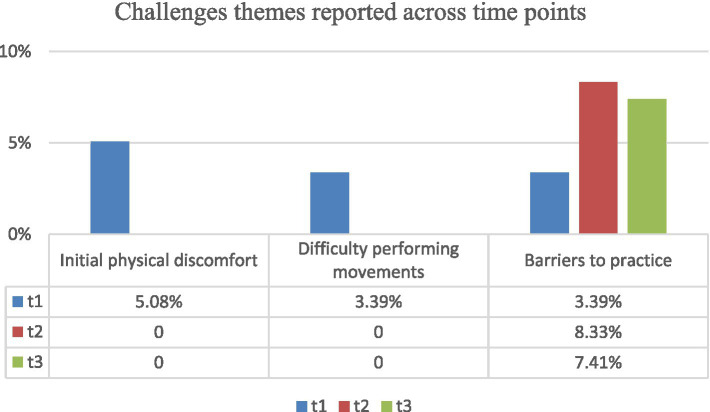
Distribution of perceived challenges related to chair yoga practice at post-intervention (t_1_), 2-week (t_2_), and three-month (t_3_) follow-up.

Qualitative themes for perceived benefits and challenges are summarized in Figures 4, 5 across the three assessment time points (t_1_, t_2_, and t_3_). Detailed information on participants’ responses across the different time points is provided in Supplementary material 2.

As shown in Figure 4, *relaxation/stress relief* was the most frequently reported benefit at all time points, decreasing from 38.98% at t_1_ to 20.83% at t_2_ and 22.22% at t_3_. Other benefits became more prominent over time: *mental calmness* and *mood improvement* were reported by 5.08 and 3.39% of participants at t_1_, both 4.17% at t_2_, and increased to 11.11% each at t_3_. *Bodily comfort* rose steadily from 5.08% (t_1_) to 12.50% (t_2_) and 14.81% (t_3_), while *physical relief* peaked at t_2_ (8.33%), 1.69% at t_1_, 3.70% at t_3_. *Sleep improvement* was reported at t_1_ (6.80%) but not at t_2_, reappearing at t_3_ (7.41%), and *self/body awareness improvement* remained relatively stable (5.08% at t_1_, 8.33% at t_2_, 7.41% at t_3_). *Transformation experiences* showed a marked transient increase at t_2_ (25.00%) compared with t_1_ (8.47%) and then declined at t_3_ (7.41%), whereas *other brief and simple positive feedbacks* were most frequent at t_1_ (13.33%) and declined at later time points (8.33% at t_2_, 7.41% at t_3_).

Figure 5 indicates that relatively few challenges were reported overall. *Initial physical discomfort* (5.08%) and *difficulty performing movements* (3.39%) were reported only at t_1_ and disappeared at t_2_ and t3. In contrast, barriers to practice emerged as the predominant ongoing challenge, increasing from 3.39% at t_1_ to 8.33% at t_2_ and remaining high at t_3_ (7.41%), suggesting that external or practical obstacles to maintaining practice persisted over time even as internally experienced difficulties diminished.

Across time points, the findings illustrate a clear progression: t_1_ reflects initial engagement with prominent relaxation benefits and notable challenges; t_2_ represents a critical adaptation phase marked by transformation experiences and increased bodily awareness; and t_3_ demonstrates consolidation, with more stable psychological and physical benefits and minimal internal challenges, though external barriers to practice remain.

## 4 Discussion

This preliminary study provides converging quantitative and qualitative evidence that a brief chair yoga intervention can meaningfully reduce psychological distress among high school teachers and that sustained practice is crucial for maintaining and deepening these benefits. The immediate post-intervention reduction in DASS-10 scores [Cohen’s d = 0.78, 95% CI (0.425, 1.125)] indicates a moderate-to-large effect (69), suggesting that even a short, five-day program can yield relevant improvements in stress reduction. Qualitative reports at this stage (t_1_) mirror these findings, with participants most frequently describing relaxation and stress relief, alongside emerging experiences of mental calmness, bodily comfort, and self/body awareness. Early mentions of physical discomfort, difficulty performing movements, and practice barriers are also in line with qualitative evaluations of school yoga and mindfulness interventions, where participants describe initial adjustment-related challenges before becoming comfortable with the practices.

At baseline, participants reported DASS-10 scores approaching the moderate range, indicating the need for feasible mental health supports in school workplaces, a need highlighted in prior research on educator stress and burnout. The significant stress reduction immediately following the chair yoga program aligns with evidence that relatively brief, structured yoga and yoga-mindfulness programs can reduce teachers’ stress (37–40, 42–45), improve affect (34, 42), and wellbeing (37–40, 42, 46–48, 57, 70, 71). Qualitatively, the predominance of relaxation and stress relief themes at t_1_ underscores that the earliest perceived gains are primarily in downregulation of tension and rapid relief from emotional strain, echoing findings from yoga interventions with teachers and education staff reporting immediate stress relief and enhanced calm. These effects are consistent with proposed mechanisms whereby yoga practices, combining gentle movement, controlled breathing, and mindful attention, modulate autonomic balance and reduce physiological arousal, thereby supporting emotion regulation and stress recovery (30, 34, 42, 47, 58, 71, 72). The presence of early-stage challenges at t_1_ suggests that while the intervention is acceptable and beneficial, teachers initially need time to become comfortable with movements, coordination, and integrating practice into busy schedules.

At the 2-week follow-up (t_2_), quantitative analyses revealed a marked divergence between teachers who continued practicing and those who did not, with a large time-practice interaction (ηp^2^ = 0.382) indicating that ongoing engagement is critical for maintaining and amplifying gains, consistent with dose–response findings in yoga and meditation research where higher practice frequency predicts greater benefits (73–78). Qualitative data at t_2_ reinforce this interpretation: although relaxation remains a prominent benefit, transformation experiences peak, and participants more often report bodily comfort and physical relief, suggesting an adaptation phase in which teachers move beyond immediate stress relief towards deeper changes in self-perception and embodied wellbeing, paralleling educator-focused yoga studies that describe increased self-awareness, resilience, and shifts in professional identity over time. Reports of mental calmness and mood improvement, though modest, indicate early consolidation of psychological benefits, in line with evidence that continued yoga and mindfulness practice enhances emotion regulation and quality of life (75, 79). The disappearance of initial physical discomfort and movement difficulty by this stage is compatible with accounts of participants adjusting to yoga postures and techniques over repeated sessions. At the same time, participants more frequently mention barriers to practice, reflecting that once novelty wanes, external constraints such as time pressure and workload become more salient (80–82).

The three-month findings (t_3_) provide particularly strong evidence for a dose–response relationship between practice frequency and psychological outcomes. Quantitatively, participants who engaged in regular practice (1–2 times per week) showed sustained and cumulative reductions in stress, reaching minimal levels at follow-up, whereas those who practiced rarely exhibited only partial maintenance, and those who did not practice experienced increases in DASS-10 scores that exceeded baseline levels, a pattern consistent with studies linking more frequent yoga or meditation practice to greater improvements in psychological wellness and stress-related outcomes (73–76, 78). The very large interaction effect (ηp^2^ = 0.537) suggests that practice behavior accounted for a substantial proportion of variance in long-term outcomes, underscoring adherence as a key determinant of intervention effectiveness, in line with broader yoga and mindfulness trials where adherence and home practice predict stronger and more durable benefits (83, 84). Although the between-subjects effect (ηp^2^ = 0.118) did not reach statistical significance, its magnitude nonetheless suggests potentially meaningful differences between groups that may have been underpowered, a situation commonly reported in small-sample yoga and mindfulness-based intervention studies (85, 86). Qualitative data at t_3_ complement this pattern: benefits become more evenly distributed, with relaxation still prominent but accompanied by higher reports of mental calmness, mood improvement, bodily comfort, self/body awareness, and sleep enhancement, mirroring qualitative accounts in educator and occupational yoga research where sustained practice is associated with broader and more stable gains in emotional balance, sleep, and coping capacity. The reduction in transformation-related language suggests that what once felt like discrete “*changes*” may have become normalized, integrated features of daily experience, similar to descriptions of yoga skills being “*taken off the mat*” and incorporated into daily routine. The persistence of external barriers to practice, despite reduced internal challenges, echoes implementation work showing that contextual constraints such as workload, time, and space remain key obstacles to long-term adoption of yoga and mindfulness practices (80–82, 87).

Across time points, the qualitative trajectory echoes and enriches the quantitative dose–response findings. T_1_ reflects initial engagement, dominated by relaxation and stress relief alongside adjustment challenges, consistent with early-stage experiences in other brief yoga-based interventions for educators. T_2_ represents a critical adaptation phase characterized by heightened transformation experiences, increasing bodily awareness and comfort, and the emergence of practical barriers to practice, in line with qualitative studies that describe both deepening psychological benefits and rising awareness of contextual obstacles as programs progress. By t_3_, the pattern suggests consolidation: psychological and physical benefits are more stable and multifaceted, internal challenges have largely receded, and external barriers, rather than acceptability or perceived value, appear to be the main obstacles to continued use, mirroring long-term follow-up findings in teacher mindfulness and yoga programs where perceived benefits persist but practice is constrained by time and institutional factors (88). This progression aligns with habit formation theory, which holds that repeated engagement in a behavior leads to increased automaticity and integration into daily routines (89), and with evidence that regular yoga practice is associated with more entrenched coping skills and psychological benefits over time (37, 45, 77, 90–92). Regular chair yoga practice may thus have shifted from being perceived as an extra activity to functioning as an accessible, embodied coping strategy that teachers draw upon to manage stress, maintain emotional balance, and buffer end-of-year occupational strain.

Integrating both strands of evidence, the data support a coherent narrative: brief, low-intensity chair yoga can produce rapid, meaningful reductions in stress; continued practice over subsequent weeks is associated with perceived transformation, deeper embodied benefits, and sustained quantitative improvement; and regular engagement over months appears to consolidate these gains into more stable patterns of psychological and physical wellbeing. At the same time, persistent reports of external barriers across t_2_ and t_3_ highlight that working conditions such as scheduling, workload, and institutional support are likely decisive for whether teachers can maintain the practice at a frequency sufficient to achieve and preserve these benefits, reinforcing implementation recommendations from school-based yoga and mindfulness research that emphasize the need for structural support to sustain educator practice (39, 93).

## 5 Implications

The present findings suggest that brief, low-intensity chair yoga can be feasibly implemented in Vietnamese high school settings and may offer a practical means of reducing stress among teachers. In a context where many teachers report pressure related to student achievement, and changing curricula yet often have limited time and access to mental health services, short, chair-based practices that can be done in the classroom or staff room are particularly well suited to everyday school routines. The clear dose–response pattern and the qualitative shift from immediate relaxation to more stable psychological and physical benefits point out the necessity of supporting teachers not only to attend initial sessions but also to maintain regular, independent practice over time.

Practically, this points to the value of embedding brief chair yoga breaks into the school day (for example, between teaching periods or after-school meetings), providing simple practice guides or videos, and encouraging peer or group practice among colleagues to enhance motivation and accountability. Moreover, organizational support such as leadership endorsement, modest timetable flexibility, provision of quiet spaces, and recognition of participation within school health or professional development plans will likely be crucial for sustaining engagement.

## 6 Limitations

This study has several limitations that should be taken into consideration. Firstly, internal validity is substantially constrained by the quasi-experimental pre–post design without a control group, absence of randomization or blinding, and exclusive reliance on self-report (DASS-10). Without a comparison condition, we cannot attribute observed changes in distress solely to the chair yoga intervention, and alternative explanations such as regression to the mean, spontaneous improvement, or unmeasured contextual changes cannot be ruled out. In addition, the brief DASS-10 may not fully capture the complexity of teachers’ mental health.

Secondly, the modest initial sample, attrition at the 2-week and three-month follow-ups, and the single-site context limit robustness and generalizability. The final analyzable sample of 41 participants from a single private semi-boarding high school also reduces statistical power and limits the generalizability of the findings beyond similar urban, private-school contexts. Attrition may also have introduced bias, favoring participants who were more motivated or more receptive to yoga, while the urban, private-school context restricts applicability to other regions, school types, or educational systems.

Thirdly, the timing of the three-month follow-up (t_3_) at the end of the academic year likely coincided with peak workload and pressure, making it difficult to disentangle end-of-year stress from genuine changes in intervention effects.

Finally, adherence and practice exposure were assessed based on self-reported categories rather than detailed or device-based monitoring, which may have introduced recall or social desirability bias and reduced the precision of dose–response estimates.

## 7 Conclusion

This preliminary study provides converging quantitative and qualitative evidence that a brief, five-day chair yoga program can meaningfully reduce psychological distress among Vietnamese high school teachers and that continued practice is essential for maintaining and deepening these gains. Immediate improvements were reflected in moderate-to-large reductions in DASS-10 scores and in participants’ reports of relaxation and stress relief, while longitudinal analyses showed a clear dose–response pattern: teachers who practiced regularly experienced the greatest and most sustained benefits, whereas those who stopped practicing showed a rebound in distress. Qualitative trajectories from early relaxation and adjustment challenges, through a phase of perceived transformation and growing bodily ease, to a more consolidated profile of psychological and physical wellbeing, paralleled these quantitative patterns and suggest that chair yoga can become a habitual coping strategy when practiced consistently. Because of the uncontrolled, single-arm design, these results should be interpreted as preliminary and non-causal, reflecting associations rather than definitive evidence that chair yoga caused the observed changes.

Within the Vietnamese educational context, where teachers face high pressure from curriculum reform, student performance expectations, and overtime teaching, these findings highlight chair yoga as a low-cost, adaptable option for supporting teacher wellbeing. However, the single-site, uncontrolled design, modest sample, attrition, and limited adherence and measurement data mean the results should be viewed as promising but preliminary. Even so, they add to international evidence that accessible school-based mind–body practices can support teacher mental health and underscore the potential for integrating chair yoga into broader efforts to promote sustainable, healthy teaching careers in Vietnam. Further research under more rigorous designs is needed to confirm these effects, clarify mechanisms, and identify the organizational and policy conditions required for scalable, long-term implementation.

## Data Availability

The raw data supporting the conclusions of this article will be made available by the authors, without undue reservation.
