# Supplementary material for: The effects of chair yoga practice on stress reduction among high school teachers in Vietnam: a preliminary quasi-experimental study
Source: Front Public Health. 2026 Jul 15;14:1867357. doi: 10.3389/fpubh.2026.1867357 (PMC13414819; doi:10.3389/fpubh.2026.1867357)
Supplement: Supplementary file 1 [file Table_1.DOCX]

Supplementary Material

# Supplementary material 1. A 5-day chair yoga program

| Day 1 | - Stabilize seated posture on the chair; - Gently warm up the legs, hands, and shoulders; - Neck stretches - Spinal warm-up (Seated Cat - Cow, circular rotations); - Seated twist; - Seated side bend; - Shoulder and arm stretches; - Seated forward fold; - Seated glute stretch; - Full yogic breathing (three-phase breath exercise). |
| --- | --- |
| Day 2 | - Stabilize seated posture on the chair; - Gently warm up the legs, hands, and shoulders; - Neck stretches - Seated twist; - Seated side bend; - Warrior II (side bend and twist) (with chair support); - Sun salutation with chair support (5 times); - High lunge with chair support; - Quadriceps stretch; - Tree pose; - Half forward fold; - Warrior III with chair support; - Full yogic breathing (three-phase breath exercise); - Lion breath exercise. |
| Day 3 | Practice similar to Day 1, with the addition of Kapalabhati. |
| Day 4 | Practice similar to Day 2, and replace the breathing exercises with the four-phase breathing; Ujjayi and Lion breath exercise. |
| Day 5 | Practice similar to Day 2, and replace the breathing exercises with the sectional breathing; Ujjayi; three-phase breathing; and Lion breath exercise. |

**Supplementary material 2. Participants feedback across time points**

| ***Themes*** | ***Representative Key Phrases*** | ***t_1_***  ***n (%)*** | ***t_2_***  ***n (%)*** | ***t_3_***  ***n (%)*** |
| --- | --- | --- | --- | --- |
| ***Benefits*** | | | | |
| Relaxation / stress relief | Feeling relaxed [*thư giãn*]  Reduce stress [*giảm căng thẳng*]  Feel comfortable/at ease [*thoải mái*] | 23 (38.98%) | 5 (20.83%) | 6 (22.22%) |
| Mental calmness | Clear head [*đầu óc nhẹ nhàng*]  Calm mind [*tâm trí yên hơn*]  Reduced rumination [*ít suy nghĩ lung tung*]  More patient [*kiên nhẫn hơn*] | 3  (5.08%) | 1 (4.17%) | 3 (11.11%) |
| Mood improvement | Happier [*Vui vẻ hơn*],  Good mood [*Tâm trạng tốt*]  Less irritable [*Ít cáu gắt hơn*]  Less anxiety [*Giảm lo lắng*] | 2  (3.39%) | 1 (4.17%) | 3 (11.11%) |
| Physical relief | Relieves pain [*giảm đau nhức*]  Relaxes muscles [*thư giãn cơ bắp*]  Relieves neck and shoulder stiffness [*Vai, cổ gáy đỡ mỏi*] | 1  (1.69%) | 2 (8.33%) | 1 (3.70%) |
| Bodily comfort | Feeling light [*nhẹ người*]  Feel comfortable/good in the body [*Cơ thể thoải mái/khoẻ khoắn*]  More engergy [*Nhiều năng lượng hơn*]  Bodily stretching [*Cơ thể được kéo giãn*]  Stable heart-rate [*Nhịp tim ổn định*] | 3  (5.08%) | 3 (12.5%) | 4 (14.81%) |
| Self/body awareness improvement | Better body awareness [*Cảm nhận cơ thể tốt hơn*]  More attention to the body [*Chú ý cơ thể nhiều hơn*]  Focus on breathing [*Tập trung vào hơi thở*] | 3  (5.08%) | 2 (8.33%) | 2 (7.41%) |
| Sleep improvement | Better sleep [*Ngủ ngon hơn*]  Less insomnia than before [*Ít bị mất ngủ như trước*]  Easier to fall as sleep [*Dễ ngủ hơn*]  Sleepy [*Buồn ngủ*] | 4  (6.80%) | 0 | 2 (7.41%) |
| Transformation | Feeling pain/tense/uncomfortable/unable to perform at first but then feel better [*Ban đầu cảm thấy đau/căng/khó chịu nhưng sau đó cảm thấy tốt hơn/thực hiện được*]  Tired but calmed the mind [*Mệt nhưng làm cho đầu óc nhẹ nhỏm*]  Better adaptation after practice time [*Thích nghi tốt hơn sau thời gian tập*] | 5  (8.47%) | 6 (25.0%) | 2 (7.41%) |
| Other brief and simple positive feedbacks | Ok [*Ổn*]  Effective [*Hiệu quả*]  Good [*Hay*]  Helpful [*Bổ ích*]  Easy to practice [*Dễ thực hiện*] | 8 (13.33%) | 2 (8.33%) | 2 (7.41%) |
| **Challenges** | | | | |
| Initial physical discomfort | Tense [*Đau mỏi*]  Muscle strain [*Căng cơ*]  Slightly uncomfortable during exercise [*Hơi khó chịu khi tập*]  Feeling exhausted after exercising [*Cảm thấy đuối sau khi tập*] | 3  (5.08%) | 0 | 0 |
| Difficulty performing movements | Difficult to perform [*khó thực hiện*],  Unfamiliar with the movements [*chưa quen với các động tác*] | 2  (3.39%) | 0 | 0 |
| Barriers to practice | Busy [*Bận rộn/ Không có thời gian*]  Too much workload [*Công việc quá nhiều*]  Short training time [*Thời gian tập luyện ít*]  Forget [*Quên*]  Difficult to maintain practice [*Khó khăn để duy trì tập luyện*] | 2  (3.39%) | 2 (8.33%) | 2 (7.41%) |
